# Eating Habits and Lifestyles during the Initial Stage of the COVID-19 Lockdown in China: A Cross-Sectional Study

**DOI:** 10.3390/nu13030970

**Published:** 2021-03-17

**Authors:** Guo-yi Yang, Xin-lei Lin, Ai-ping Fang, Hui-lian Zhu

**Affiliations:** Guangdong Provincial Key Laboratory of Food, Nutrition and Health, Department of Nutrition, School of Public Health, Sun Yat-Sen University, Guangzhou 510080, China; yanggy@connect.hku.hk (G.-y.Y.); linxlei3@mail2.sysu.edu.cn (X.-l.L.); fangaip@mail.sysu.edu.cn (A.-p.F.)

**Keywords:** COVID-19, eating habits, lifestyles, lockdown

## Abstract

Due to the outbreak of coronavirus disease 2019 (COVID-19), the Chinese government implemented strict lockdown measures to control the spread of infection. The impact of the COVID-19 lockdown on eating habits and lifestyles in the general population is unclear. This cross-sectional study was conducted via an online survey to obtain an overview of the food access, food intake, and physical activity of Chinese residents during the initial stage of the COVID-19 lockdown, and to investigate the association between staying at home/working from home and changes in eating habits and lifestyles. A total of 2702 participants (70.7% women) were included. Most of the participants maintained their habitual diet, while 38.2% increased their snack intake, 54.3% reported reduced physical activity, and 45.5% had increased sleep duration. Most people (70.1%) reported no change in body weight, while 25.0% reported an increase. Always staying at home/working from home was associated with an increase in animal product, vegetable, fruit, mushroom, nut, water, and snack intake, as well as sleep duration and frequency of skipping breakfast (odds ratio (OR) 1.54, 1.62, 1.58, 1.53, 1.57, 1.52, 1.77, 2.29, and 1.76 respectively). Suggestions should be made to encourage people to reduce their snack intake, maintain the daily consumption of breakfast, and increase physical activity during future lockdown periods.

## 1. Introduction

Coronavirus diseases 2019 (COVID-19), caused by the severe acute respiratory syndrome coronavirus 2 (SARS-CoV-2) [1], emerged rapidly in China in December 2019 [2]. In response to the spread of the virus, the Chinese government implemented strict measures such as the restriction of transportation, closure of public places, and home confinement. Chinese residents were required to reduce unnecessary outdoor activities from 23 January, when the lockdown in Wuhan was officially announced. Most people stayed at home or worked from home, while others returned to work during the lockdown period, with great effects on people’s habitual lifestyles.

Previous studies have suggested an increase in anxiety and depressive symptoms among the general population during the COVID-19 pandemic [3]. Home confinement causes boredom and isolation, and can aggravate anxiety and stress [4]. Such negative impacts on psychological health could provoke the overeating of unhealthy food and result in weight gain [5,6,7,8]. However, due to the suspension of transportation in addition to economic decline and home confinement, access to food may have been limited during the lockdown, possibly leading to food insecurity and reduced food intake [9,10,11].

Nutrition, which can exert anti-inflammatory and immunomodulatory effects, is essential to reduce susceptibility to COVID-19 and mitigate potential complications [12,13]. Despite the importance of maintaining healthy diets and lifestyles during the pandemic, the impact of COVID-19 lockdown on eating habits and lifestyles in the general population is unclear. Therefore, this cross-sectional study was conducted via an online survey to obtain an overview of the food access, food intake, and physical activity of Chinese residents during the initial stage of the COVID-19 lockdown, and to investigate the association of staying at home/working from home with changes in eating habits and lifestyles.

## 2. Methods

### 2.1. Study Design and Participants

An online survey was conducted via the questionnaire platform “Wenjuan xing” (Wenjuan xing Tech Co. Ltd., Changsha, China), and was distributed via “WeChat” (Tencent Inc., Shenzhen, China) between 23 February and 4 March 2020. A convenience sampling method was used. To determine whether participants answered the questions carefully, the time spent completing the whole questionnaire was recorded. Participants aged <18 years old or participants who completed questionnaires within 3 min were excluded.

### 2.2. Data Collection

The anonymous questionnaire consisted of questions about sociodemographic characteristics, anthropometrics, food access, food intake, physical activity, and changes in eating habits and lifestyles during the COVID-19 lockdown. As the Wuhan lockdown was officially announced on 23 January, the COVID-19 lockdown period was defined as the month after 23 January, while the pre-COVID-19 period was defined as the 3 months prior to 23 January in the questionnaire. The questionnaire was evaluated by both experienced researchers in the field of sports and nutrition and a non-expert group to ensure the simplicity and clarity of the questions.

### 2.3. Sociodemographic Characteristics and Anthropometrics

Data on self-reported age, sex, height, weight, educational levels, occupation, chronic disease history, and status during lockdown were collected. Educational levels were categorized as: “secondary or below”, “college”, and “postgraduate or above”. The occupation categories were as follows: “medical worker”, “civil servant”, “farmer/factory worker”, “enterprise worker”, “researcher”, “student”, and “others”. Status during lockdown was classified depending on whether the participants had returned to work within the first week, within the second week, or within the third week after the lockdown announced (i.e., 24–30 January, 31 January–6 February or 7–13 February, respectively), or had always stayed at home/worked from home. Body mass index (BMI) was calculated as weight by height squared (kg/m^2^).

### 2.4. Food Access and Food Intake during the COVID-19 Lockdown

The frequencies of shopping in person, ordering food online, and eating out were classified as “never”, “sometimes”, or “often”. Information was collected on the amount of consumption per instance and the frequency of consumption for different kinds of foods, i.e., rice, noodles, stuffed buns, whole grain food, livestock meat, poultry meat, aquatic products, eggs, leaf vegetables, melon/solanaceous vegetables, fruits, mushrooms, nuts, milk, yogurt, beans, tofu, soybean milk, and water. The weights and volumes of common cooked foods were provided in the questionnaire to guide the participants in estimating the amount of food consumed per instance. Food intakes were approximated as the amount per instance multiplied by the frequency of consumption (g/day or mL/day).

### 2.5. Physical Activity during the COVID-19 Lockdown

Data on the frequency and the duration of low-intensity, moderate-intensity, and vigorous-intensity physical activity during the COVID-19 lockdown were collected. Different examples of physical activity were provided to guide the participants in identifying their physical activity levels. The amount of time spent doing different levels of physical activity per week were approximated as the duration multiplied by the frequency of physical activity (min/week). Total weekly physical activity was estimated by adding up the weekly time of each level of physical activity.

### 2.6. Changes in Eating Habits and Lifestyles during the COVID-19 Lockdown

Changes in eating habits and lifestyles were classified as “decreased”, “unchanged”, or “increased” during the COVID-19 lockdown as compared with the pre-COVID-19 period. Changes in the consumption of staple foods, animal products, vegetables, fruits, mushroom, nuts, dairy products, legumes, water, and snacks were collected. Changes in physical activity, frequency of eating breakfast and midnight snacks, sleep duration, and body weight were also included in the questionnaire.

### 2.7. Statistical Analysis

Values were reported as the mean ± standard deviation (SD) for continuous variables with normal distribution, as the median (interquartile range (IQR)) for continuous variables with skewed distribution, or as the frequency (percentage) for categorical variables. Differences in food access, food intake, and physical activity during COVID-19 lockdown by status during lockdown were analyzed using Kruskal–Wallis H tests, where post hoc comparisons were adjusted by Bonferroni corrections. Chi-squared tests were used to analyze changes in eating habits and lifestyles during the COVID-19 lockdown by status. Multinomial logistic regression was used to access the association of status during the lockdown with changes in eating habits and lifestyles, adjusted for age, sex, BMI, and educational levels. Status during lockdown was grouped into 4 categories (i.e., returned to work within the first week, within the second week, or within the third week after the lockdown was announced, or always stayed at home/worked from home), where the group that returned to work within the first week was set as the comparison group. Statistical analysis was performed using SPSS 23.0. The significance level was set at two-sided *p* < 0.05.

## 3. Results

### 3.1. Sociodemographic Characteristics and Anthropometrics of Participants

A total of 2723 Chinese residents participated in the online survey. All participants completed the questionnaire for more than 3 minutes. Participants aged <18 years old were excluded (*n* = 11), and finally 2702 participants (70.7% women) were included in the analysis. Participants spent an average of 8.8 (6.7–12.2) minutes filling in the questionnaire. Among the participants, 68.9% were between 18 and 44 years old, 60.7% had a college degree, 66.9% had a BMI between 18.5 and 23.9 kg/m^2^, and 84.3% had no history of chronic disease. The survey covered all 34 provincial-level administrative regions in China, where most participants (67.9%) lived in Guangdong Province during the lockdown period. The sociodemographic characteristics and anthropometrics of participants by status during lockdown are presented in Table 1.

### 3.2. Food Access during the COVID-19 Lockdown

A total of 69.4% participants reported sometimes or often shopping in person during the COVID-19 lockdown. Thirty percent of participants ordered food online, and 6.4% reported eating out during the lockdown. Data on food access by status during the lockdown are presented in Table 2. Participants who always stayed at home/worked from home were less likely to go shopping in person or order food online than those who returned to work during within the first week or within the second week after the lockdown (all *p* values for post hoc comparisons <0.05).

### 3.3. Food Intake during the COVID-19 Lockdown

Food intake during the COVID-19 lockdown is summarized in Table 3. There were significant differences in the consumption of poultry meat, aquatic products, fruits, milk, yogurt, and water during the COVID-19 lockdown by status. Participants who always stayed at home/worked from home tended to consume fewer aquatic products but more fruits than those who returned to work within the first week (17.2 vs. 17.2 g/day and 150.0 vs. 85.8 g/day, respectively), ate less poultry meat, milk, and yogurt than those who returned to work within the second week (20.0 vs. 32.9 g/day, 50.0 vs. 71.5 g/day and 14.3 vs. 35.7 g/day, respectively) and ate less poultry meat than those who returned to work within the third week (20.0 vs. 32.9 g/day).

### 3.4. Physical Activity during the COVID-19 Lockdown

Different levels of physical activity during the COVID-19 lockdown are shown in Table 4. The amount of time spent performing physical activity per week were 45.0 (3.8–157.5) mins, 3.8 (3.8–45.0) mins, and 3.8 (3.8–3.8) mins for low, moderate, and vigorous intensity, respectively. The total weekly time spent in physical activity was 105.0 (22.5–281.3) mins. There was no significant difference in the time spent performing each level of physical activity or total physical activity by status during the lockdown.

### 3.5. Changes in Eating Habits and Lifestyles during the COVID-19 Lockdown

The changes in eating habits and lifestyles during COVID-19 lockdown as compared with the pre-COVID-19 period are summarized in Table 5. Most of the participants reported no changes in the consumption of different kinds of foods (i.e., staple foods, animal products, vegetables, fruits, mushroom, nuts, dairy products, legumes, water, and snacks) and the frequencies of having breakfast or midnight snacks. However, 38.2% of the participants reported an increase in snack intake. There were 54.3% of the participants who reported reduced physical activity and 45.5% who increased their sleep duration during the COVID-19 lockdown. The majority of the participants (70.1%) reported no change in body weight, while 25.0% reported an increase and 4.9% reported a decrease.

The results of the multinomial logistic regression of status during lockdown on changes in eating habits and lifestyles are shown in Table 6. Staying at home/working from home was associated with an increase in the intake of animal products, vegetables, fruits, mushrooms, nuts, water, and snacks (odds ratio (OR) 1.54, 1.62, 1.58, 1.53, 1.57, 1.52, and 1.77, respectively), and was associated with a decrease in dairy product intake (OR 1.85). Compared with participants who returned to work within the first week, participants who always stayed at home/worked from home were more likely to either show an increase or decrease in physical activity (OR 1.69 for “increased” vs. “unchanged” and 1.44 for “decreased” vs. “unchanged”). Besides, participants who always stayed at home/worked from home were more likely to skip breakfast and increase their sleep duration during the COVID-19 lockdown (OR 1.76 and 2.29, respectively), as compared with those who returned to work within the first week. *p* values for model fitting were all <0.001.

## 4. Discussion

Our study obtained an overview in relation to the food access, food intake, and physical activity of Chinese residents during the initial stage of the COVID-19 lockdown. Most of the participants maintained their habitual diets but had reduced physical activity during the lockdown. Always staying at home/working from home was associated with an increase in the intake of animal products, vegetables, fruits, mushroom, nuts, water, and snacks, and with a decrease in dairy product intake during the COVID-19 lockdown.

Despite home confinement, most of the participants obtained food via shopping in person during the lockdown. Some participants ordered food online, while few people reported eating out, possibly due to the closure of most restaurants during the initial stage of lockdown. Participants who stayed at home/worked from home were less likely to go shopping in person or order food online, which suggested that they may spend more time cooking at home. López-Moreno et al. showed that 73.5% people reported better cooking at home in a Spanish population during COVID-19 home confinement [14]. Similarly, Ruiz-Roso et al. found that families had more time to cook at home during the pandemic [15].

The majority of the participants declared no change in the intake of staple foods, animal products, vegetables, fruits, mushrooms, nuts, dairy products, legumes, water, and snacks during the COVID-19 lockdown. The results indicate that basic food supplies were guaranteed in China during the initial stage of the lockdown. These findings were consistent with another cross-sectional study among adults in the Netherlands, which showed that 83% of participants did not change their eating habits during the COVID-19 lockdown [16].

Notably, 38.2% people reported increased snack intake during the lockdown, which was the most pronounced change found among all kinds of foods. The result is in line with those reported by Di Renzo et al. and Ammar et al., who reported an increase in snack and unhealthy food intake during the COVID-19 lockdown [17,18]. This may have been driven by anxiety, depression, or boredom during the lockdown. Wang et al. reported that more than half of the respondents rated the psychological impact as moderate–severe, and one-third reported moderate–severe anxiety during the initial stage of the COVID-19 outbreak in China [19]. Such negative psychological impacts could lead to emotional eating and sweet cravings [7,20].

Staying at home/working from home was associated with an increase in the intake of fruits and vegetables. Most of the dietary recommendations encouraged the consumption of fruits and vegetables during the pandemic [21]. Fresh fruits and vegetables contain large amounts of vitamins and minerals including vitamins A, C, and D, as well as selenium and zinc, which enhance immunity and prevent virus infection [22,23]. Staying at home/working from home was also associated with an increase in the consumption of animal products, mushrooms, nuts, and snacks. Similarly, Scarmozzino et al. showed that more than half of the respondents reported eating more during the confinement in an Italian sample [24]. The “Effects of home Confinement on multiple Lifestyle Behaviours during the COVID-19 outbreak” (ECLB-COVID19) International Online Survey reported that participants were more likely to eat out of control during the confinement period than before [18]. It is possible that people may have eaten out of anxiety or boredom when spending more time at home during the lockdown [6,8].

More than half of the participants reported a decline in physical activity during the COVID-19 lockdown. The results may be attributed to the closure of gyms and sports centers, the restriction of access to public places, and the lack of space and facilities at home for exercise [25]. The finding is consistent with those reported by Ammar et al., who found that people had reduced levels of physical activity and increased sedentary hours during the COVID-19 pandemic [18]. The amount of physical activity drastically decreased, while dietary intakes remained unchanged or increased during home confinement, possibly leading to a positive energy balance. Short-term physical inactivity and a positive energy balance have negative health consequences in relation to reduced insulin sensitivity, higher total body fat and central fat, and a proinflammatory state [26]. Physical activity enhances immune functions against infection [27], improves mental health, and results in healthier food choices [28]. The practice of physical exercise at home, such as jogging, rope skipping, and high-intensity interval training (HIIT), should be recommended to promote psychological and physical health during the period of confinement [29,30].

Despite the decrease in physical activity, most participants maintained their body weight during the lockdown. However, 25.0% reported an increase in body weight. The proportion of self-reported weight gain is smaller here than shown in Poland and Italy [17,31], where 29.9% and 48.6% participants reported weight gain, respectively. Given that obesity is associated with a severe clinical course of COVID-19 and increased mortality from infection [32,33], strategies to control body weight during lockdown periods should be considered.

Nearly half of the participants declared an increase in sleep duration during the COVID-19 lockdown. Likewise, Trakada et al. found that the self-reported sleep duration increased in general population during the pandemic, but one-third of respondents reported worse sleep quality than in normal situations [34]. Those who had always stayed at home/worked from home were more likely to increase their sleep duration and skip breakfast during the lockdown. The daily consumption of breakfast improves appetite and satiety and reduces unhealthy snacking behaviors, improving dietary quality [35]. Thus, suggestions to encourage the consumption of breakfast should be emphasized to promote a balanced diet during the COVID-19 lockdown.

To our knowledge, few studies have investigated eating habits and lifestyles in the general population in China during the initial stage of the COVID-19 lockdown. Our study had a large sample size of more than 2700 participants. The data were collected within 10 days after the first month of the lockdown period to reduce recall bias. Nevertheless, there are some limitations of this study. First, eating habits and lifestyles before the COVID-19 lockdown were not investigated in order to avoid an excessively long questionnaire. Thus, only food intakes and perceived changes in eating habits and lifestyles during the lockdown were reported. Secondly, most of the participants in our study were women aged 18–44 years with an educational level of college or above, possibly due to voluntary sampling and the use of an Internet platform [36,37]. Selection bias should not be underestimated in the interpretation of these results. Thirdly, we cannot exclude the possibility of misreporting caused by the use of a self-reported questionnaire. Fourthly, this study should be considered as providing preliminary results on eating habits and lifestyles of Chinese residents during COVID-19 lockdown. Indices of dietary quality [38] such as the Healthy Eating Index (HEI) and the Diet Quality Index (DQI) were not used. Further investigations are needed to assess the long-term effects of home confinement on dietary quality and health outcomes during the COVID-19 pandemic.

## 5. Conclusions

This study provides an overview of the food access, food intake, and physical activity of Chinese residents during the initial stage of the COVID-19 lockdown. Most people obtained food via shopping in person or by ordering food online during the lockdown. The majority of participants maintained their eating habits, while 38.2% increased their snack intake. Most people had reduced physical activity and increased sleep duration during the lockdown.

Always staying at home/working from home was associated with an increase in food consumption, sleep duration, and frequency of skipping breakfast. Recommendations should be made to encourage people to reduce snack intake, maintain the daily consumption of breakfast, and increase physical activity during future lockdown periods.

## Figures and Tables

**Table 1:** Participants´ general characteristics and anthropometrics ^1^.

Variables	Whole Participants	Returned to Work within the First Week	Returned to Work within the Second Week	Returned to Work within the Third Week	Always Stayed at Home/Worked from Home
	(*n* = 2702)	(*n* = 455)	(*n* = 297)	(*n* = 298)	(*n* = 1652)
Age (year)	37.3 ± 12.0	40.2 ± 10.7	38.3 ± 11.0	36.5 ± 10.7	36.4 ± 12.6
Age groups (year)					
18–44	1862 (68.9)	268 (58.9)	196 (66.0)	222 (74.5)	1176 (71.2)
45–59	766 (28.3)	181 (39.8)	99 (33.3)	75 (25.2)	411 (24.9)
≥60	74 (2.7)	6 (1.3)	2 (0.7)	1 (0.3)	65 (3.9)
Sex					
Men	793 (29.3)	150 (33.0)	79 (26.6)	108 (36.2)	456 (27.6)
Women	1909 (70.7)	305 (67.0)	218 (73.4)	190 (63.8)	1196 (72.4)
Height (m)	163.4 ± 7.4	163.0 ± 7.5	163.2 ± 7.2	164.0 ± 7.2	163.4 ± 7.4
Weight (kg)	58.7 ± 10.2	59.5 ± 10.3	59.2 ± 10.4	58.8 ± 10.1	58.3 ± 10.1
BMI (kg/m^2^)	21.9 ± 2.8	22.3 ± 2.8	22.1 ± 2.8	21.8 ± 2.9	21.8 ± 2.9
BMI groups (kg/m^2^)					
<18.5	283 (10.5)	37 (8.1)	21 (7.1)	39 (13.1)	186 (11.3)
18.5–23.9	1808 (66.9)	299 (65.7)	203 (68.4)	193 (64.8)	1113 (67.4)
≥24	611 (22.6)	119 (26.2)	73 (24.6)	66 (22.1)	353 (21.4)
Educational level					
Secondary or below	275 (10.2)	44 (9.7)	12 (4.0)	13 (4.4)	206 (12.5)
College	1641 (60.7)	289 (63.5)	165 (55.6)	185 (62.1)	1002 (60.7)
Postgraduate or above	786 (29.1)	122 (26.8)	120 (40.4)	100 (33.6)	444 (26.9)
Occupation					
Medical worker	610 (22.6)	255 (56.0)	112 (37.7)	50 (16.8)	193 (11.7)
Civil servant	427 (15.8)	106 (23.3)	53 (17.8)	32 (10.7)	236 (14.3)
Farmer/factory worker	111 (4.1)	21 (4.6)	6 (2.0)	8 (2.7)	76 (4.6)
Enterprise worker	647 (23.9)	28 (6.2)	71 (23.9)	133 (44.6)	415 (25.1)
Researcher	110 (4.1)	3 (0.7)	14 (4.7)	14 (4.7)	79 (4.8)
Student	481 (17.8)	15 (3.3)	30 (10.1)	44 (14.8)	392 (23.7)
Others	316 (11.7)	27 (5.9)	11 (3.7)	17 (5.7)	261 (15.8)
History of chronic disease					
Yes	425 (15.7)	71 (15.6)	56 (18.9)	50 (16.8)	248 (15.0)
No	2277 (84.3)	384 (84.4)	241 (81.1)	248 (83.2)	1404 (85.0)

^1^ Values are reported using the means ± standard deviation (SD) for continuous variables or frequency (percentage) for categorical variables. BMI: body mass index.

**Table 2:** Food access during the COVID-19 lockdown by status ^1^.

Variables	Whole Participants	Returned to Work within the First Week	Returned to Work within the Second Week	Returned to Work within the Third Week	Always Stayed at Home/Worked from Home	*p* Value ^2^
	(*n* = 2702)	(*n* = 455)	(*n* = 297)	(*n* = 298)	(*n* = 1652)	
Shopping in person						<0.001
Never	827 (30.6)	111 (24.4)	58 (19.5)	83 (27.9)	575 (34.8) ^†,‡^	
Sometimes	1389 (51.4)	232 (51.0)	169 (56.9)	158 (53.0)	830 (50.2)	
Often	486 (18.0)	112 (24.6)	70 (23.6)	57 (19.1)	247 (15.0)	
Ordering food online						<0.001
Never	1891 (70.0)	282 (62.0)	188 (63.3)	213 (71.5) ^†^	1208 (73.1) ^†,‡^	
Sometimes	706 (26.1)	136 (29.9)	94 (31.6)	77 (25.8)	399 (24.2)	
Often	105 (3.9)	37 (8.1)	15 (5.1)	8 (2.7)	45 (2.7)	
Eating out						0.093
Never	2528 (93.6)	421 (92.5)	272 (91.6)	274 (91.9)	1561 (94.5)	
Sometimes	153 (5.7)	27 (5.9)	21 (7.1)	24 (8.1)	81 (4.9)	
Often	21 (0.8)	7 (1.5)	4 (1.3)	0 (0.0)	10 (0.6)	

^1^ Values are reported using the frequency (percentage). ^2^ *p* values were calculated using Kruskal–Wallis H tests, where post hoc comparisons were adjusted by Bonferroni corrections. ^†^ Different from participants who returned to work within the first week; ^‡^ Different from participants who returned to work within the second week.

**Table 3:** Food intake during the COVID-19 lockdown by status ^1^.

Variables	Whole Participants	Returned to Work within the First Week	Returned to Work within the Second Week	Returned to Work within the Third Week	Always Stayed at Home/Worked from Home	*p* Value ^2^
	(*n* = 2702)	(*n* = 455)	(*n* = 297)	(*n* = 298)	(*n* = 1652)	
Rice (g/day)	182.1 (100.0–300.0)	300.0 (100.0–300.0)	150.0 (100.0–300.0)	257.1 (100.0–300.0)	150.0 (100.0–300.0)	0.217
Noodles (g/day)	42.9 (14.3–100.0)	42.9 (10.7–85.8)	42.9 (14.3–85.8)	42.9 (14.3–85.8)	42.9 (14.3–107.1)	0.064
Stuffed buns (g/day)	14.3 (3.6–44.7)	14.3 (3.6–42.9)	14.3 (3.6–42.9)	14.3 (3.6–50.0)	14.3 (3.6–42.9)	0.514
Whole grain food (g/day)	14.3 (3.6–50.0)	14.3 (3.6–50.0)	14.3 (3.6–50.0)	14.3 (3.6–42.9)	14.3 (3.6–50.0)	0.141
Livestock meat (g/day)	42.8 (20.0–82.1)	42.8 (20.0–82.1)	60.0 (20.0–115.0)	60.0 (20.0–115.0)	42.8 (17.2–82.1)	0.090
Poultry meat (g/day)	32.9 (12.4–60.0)	32.9 (14.3–60.0)	32.9 (17.2–60.0)	32.9 (17.2–60.0)	20.0 (5.7–60.0) ^‡,§^	0.001
Aquatic products (g/day)	17.2 (4.3–40.0)	17.2 (4.3–42.8)	17.2 (5.7–42.8)	17.2 (5.4–40.7)	17.2 (4.3–40.0) ^†^	0.010
Eggs (g/day)	42.8 (17.2–60.0)	42.8 (17.2–60.0)	60.0 (20.0–60.0)	42.8 (17.2–60.0)	42.8 (17.2–60.0)	0.159
Leaf vegetables (g/day)	150.0 (75.0–300.0)	150.0 (75.0–300.0)	150.0 (75.0–300.0)	150.0 (75.0–300.0)	150.0 (75.0–300.0)	0.064
Melon/solanaceous vegetables (g/day)	53.6 (21.5–114.4)	53.6 (21.5–107.1)	53.6 (21.5–150.0)	75.0 (21.5–107.1)	75.0 (21.5–150.0)	0.120
Fruits (g/day)	107.1 (50.0–214.2)	85.8 (42.9–150.0)	107.1 (51.8–214.2)	107.1 (53.6–150.0)	150.0 (53.6–300.0) ^†^	0.013
Mushroom (g/day)	10.7 (2.7–17.9)	10.7 (2.7–17.9)	10.7 (2.7–17.9)	8.9 (2.7–17.9)	10.7 (2.7–17.9)	0.725
Nuts (g/day)	10.7 (2.7–26.8)	10.7 (2.7–25.0)	10.7 (0.9–26.8)	10.7 (2.7–25.5)	10.7 (2.7–26.8)	0.316
Milk (mL/day)	71.5 (10.7–150.0)	71.5 (14.3–150.0)	71.5 (14.3–178.5)	42.9 (10.7–150.0) ^‡^	50.0 (10.7–150.0) ^‡^	0.002
Yogurt (mL/day)	17.8 (3.6–71.5)	17.8 (3.6–71.5)	35.7 (3.6–100.0)	28.4 (3.6–71.5)	14.3 (3.6–71.5) ^‡^	0.011
Beans (times/week)	2.0 (0.5–2.0)	2.0 (0.5–2.0)	2.0 (0.5–2.0)	2.0 (0.5–2.0)	2.0 (0.5–2.0)	0.942
Tofu (times/week)	2.0 (0.5–2.0)	2.0 (0.5–2.0)	2.0 (0.5–2.0)	2.0 (0.5–2.0)	2.0 (0.5–2.0)	0.486
Soybean milk (times/week)	0.5 (0.5–2.0)	0.5 (0.5–2.0)	0.5 (0.5–2.0)	0.5 (0.5–2.0)	0.5 (0.5–2.0)	0.066
Water (mL/day)	1250 (750–1750)	1250 (750–1750)	1250 (750–1750)	1250 (750–1750)	1250 (750–1750)	0.034

^1^ Values are reported using the median (interquartile range (IQR)). ^2^ *p* values were calculated using Kruskal–Wallis H tests, where post hoc comparisons were adjusted by Bonferroni corrections. ^†^ Different from participants who returned to work within the first week; ^‡^ Different from participants who returned to work within the second week; ^§^ Different from participants who returned to work within the third week.

**Table 4:** Different levels of physical activity during the COVID-19 lockdown by status ^1^.

Variables	Whole Participants	Returned to Work within the First Week	Returned to Work within the Second Week	Returned to Work within the Third Week	Always Stayed at Home/Worked from Home	*p* Value ^2^
	(*n* = 2702)	(*n* = 455)	(*n* = 297)	(*n* = 298)	(*n* = 1652)	
Low intensity (min/week)	45.0 (3.8–157.5)	45.0 (3.8–157.5)	52.5 (3.8–157.5)	45.0 (3.8–157.5)	45.0 (3.8–157.5)	0.431
Moderate intensity (min/week)	3.8 (3.8–45.0)	3.8 (3.8–45.0)	3.8 (3.8–45.0)	3.8 (3.8–45.0)	3.8 (3.8–45.0)	0.506
Vigorous intensity (min/week)	3.8 (3.8–3.8)	3.8 (3.8–15.0)	3.8 (3.8–3.8)	3.8 (3.8–3.8)	3.8 (3.8–3.8)	0.123
Total physical activity (min/week)	105.0 (22.5–281.3)	97.5 (18.8–315.0)	120.0 (24.4–315.0)	97.5 (22.5–232.5)	108.8 (18.8–292.5)	0.383

^1^ Values are reported using the median (interquartile range (IQR)). ^2^ *p* values were calculated using Kruskal–Wallis H tests.

**Table 5:** Changes in eating habits and lifestyles during the COVID-19 lockdown by status ^1^.

Variables	Whole Participants	Returned to Work within the First Week	Returned to Work within the Second Week	Returned to Work within the Third Week	Always Stayed at Home/Worked from Home	*p* Value ^2^
	(*n* = 2702)	(*n* = 455)	(*n* = 297)	(*n* = 298)	(*n* = 1652)	
Staple food						0.112
Decreased	351 (13.0)	56 (12.3)	26 (8.8)	33 (11.1)	236 (14.3)	
Unchanged	1844 (68.2)	323 (71.0)	211 (71.0)	204 (68.5)	1106 (66.9)	
Increased	507 (18.8)	76 (16.7)	60 (20.2)	61 (20.5)	310 (18.80)	
Animal products						0.015
Decreased	471 (17.4)	76 (16.7)	46 (15.5)	45 (15.1)	304 (18.4)	
Unchanged	1714 (63.4)	315 (69.2)	199 (67.0)	187 (62.8)	1013 (61.3)	
Increased	517 (19.1)	64 (14.1)	52 (17.5)	66 (22.1)	335 (20.3)	
Vegetables						0.001
Decreased	316 (11.7)	62 (13.6)	42 (14.1)	40 (13.4)	172 (10.4)	
Unchanged	1702 (63.0)	311 (68.4)	187 (63.0)	182 (61.1)	1022 (61.9)	
Increased	684 (25.3)	82 (18.0)	68 (22.9)	76 (25.5)	458 (27.7)	
Fruits						0.023
Decreased	483 (17.9)	78 (17.1)	53 (17.8)	54 (18.1)	298 (18.0)	
Unchanged	1481 (54.8)	282 (62.0)	166 (55.9)	157 (52.7)	876 (53.0)	
Increased	738 (27.3)	95 (20.9)	78 (26.3)	87 (29.2)	478 (28.9)	
Mushroom						0.006
Decreased	515 (19.1)	83 (18.2)	50 (16.8)	70 (23.5)	312 (18.9)	
Unchanged	1850 (68.5)	330 (72.5)	216 (72.7)	179 (60.1)	1125 (68.1)	
Increased	337 (12.5)	42 (9.2)	31 (10.4)	49 (16.4)	215 (13.0)	
Nuts						0.012
Decreased	361 (13.4)	70 (15.4)	30 (10.1)	45 (15.1)	216 (13.1)	
Unchanged	1631 (60.4)	294 (64.6)	190 (64.0)	168 (56.4)	979 (59.3)	
Increased	710 (26.3)	91 (20.0)	77 (25.9)	85 (28.5)	457 (27.7)	
Dairy products						<0.001
Decreased	579 (21.4)	64 (14.1)	60 (20.2)	66 (22.1)	389 (23.5)	
Unchanged	1663 (61.5)	320 (70.3)	190 (64.0)	186 (62.4)	967 (58.5)	
Increased	460 (17.0)	71 (15.6)	47 (15.8)	46 (15.4)	296 (17.9)	
Legumes						0.024
Decreased	676 (25.0)	95 (20.9)	66 (22.2)	78 (26.2)	437 (26.5)	
Unchanged	1713 (63.4)	320 (70.3)	197 (66.3)	183 (61.4)	1013 (61.3)	
Increased	313 (11.6)	40 (8.8)	34 (11.4)	37 (12.4)	202 (12.2)	
Water						0.031
Decreased	434 (16.1)	68 (14.9)	43 (14.5)	51 (17.1)	272 (16.5)	
Unchanged	1537 (56.9)	290 (63.7)	174 (58.6)	157 (52.7)	916 (55.4)	
Increased	731 (27.1)	97 (21.3)	80 (26.9)	90 (30.2)	464 (28.1)	
Snacks						<0.001
Decreased	367 (13.6)	63 (13.8)	28 (9.4)	35 (11.7)	241 (14.6)	
Unchanged	1304 (48.3)	267 (58.7)	150 (50.5)	127 (42.6)	760 (46.0)	
Increased	1031 (38.2)	125 (27.5)	119 (40.1)	136 (45.6)	651 (39.4)	
Exercise						0.027
Decreased	1467 (54.3)	228 (50.1)	163 (54.9)	158 (53.0)	918 (55.6)	
Unchanged	904 (33.5)	184 (40.4)	96 (32.3)	103 (34.6)	512 (31.5)	
Increased	331 (12.3)	43 (9.5)	38 (12.8)	37 (12.4)	213 (12.9)	
Breakfast frequency						<0.001
Decreased	638 (23.6)	71 (15.6)	53 (17.8)	78 (26.2)	436 (26.4)	
Unchanged	1930 (71.4)	361 (79.3)	231 (77.8)	203 (68.1)	1135 (68.7)	
Increased	134 (5.0)	23 (5.1)	13 (4.4)	17 (5.7)	81 (4.9)	
Midnight snack frequency						0.679
Decreased	426 (15.8)	68 (14.9)	40 (13.5)	45 (15.1)	273 (16.5)	
Unchanged	2052 (75.9)	355 (78.0)	233 (78.5)	229 (76.8)	1235 (74.8)	
Increased	224 (8.3)	32 (7.0)	24 (8.1)	24 (8.1)	144 (8.7)	
Sleep duration						<0.001
Decreased	257 (9.5)	99 (21.8)	28 (9.4)	19 (6.4)	111 (6.7)	
Unchanged	1216 (45.0)	240 (52.7)	137 (46.1)	127 (42.6)	712 (43.1)	
Increased	1229 (45.5)	116 (25.5)	132 (44.4)	152 (51.0)	829 (50.2)	
Body weight						0.167
Decreased	122 (4.9)	30 (6.8)	13 (4.7)	11 (4.0)	68 (4.6)	
Unchanged	1744 (70.1)	313 (71.1)	186 (66.7)	187 (68.0)	1058 (70.9)	
Increased	621 (25.0)	97 (22.0)	80 (28.7)	77 (28.0)	367 (24.6)	

^1^ Values are reported using the frequency (percentage). ^2^ *p* values were calculated using chi-squared tests.

**Table 6:** Multinomial logistic regression of status during lockdown on changes in eating habits and lifestyles ^1^.

Variation		Returned to Work within the Second Week			Returned to Work within the Third Week			Always Stayed at Home/Worked from Home		
		OR	95% CI	*p* Value	OR	95% CI	*p* Value	OR	95% CI	*p* Value
Staple food	“Decreased” vs. “unchanged”	0.66	0.40–1.08	0.101	0.89	0.56–1.43	0.638	1.18	0.86–1.63	0.302
	“Increased” vs. “unchanged”	1.08	0.73–1.59	0.696	1.20	0.81–1.76	0.364	1.09	0.82–1.45	0.545
Animal products	“Decreased” vs. “unchanged”	0.94	0.62–1.41	0.762	0.97	0.64–1.47	0.904	1.19	0.90–1.59	0.219
	“Increased” vs. “unchanged”	1.18	0.79–1.78	0.421	1.61	1.09–2.38	0.018	1.54	1.14–2.08	0.005
Vegetables	“Decreased” vs. “unchanged”	1.05	0.68–1.62	0.825	1.00	0.64–1.55	0.988	0.79	0.57–1.09	0.153
	“Increased” vs. “unchanged”	1.29	0.89–1.87	0.182	1.50	1.04–2.17	0.029	1.62	1.24–2.12	<0.001
Fruits	“Decreased” vs. “unchanged”	1.06	0.71–1.59	0.778	1.13	0.76–1.69	0.550	1.09	0.82–1.45	0.565
	“Increased” vs. “unchanged”	1.29	0.90–1.85	0.159	1.60	1.12–2.27	0.010	1.58	1.21–2.05	0.001
Mushroom	“Decreased” vs. “unchanged”	0.89	0.60–1.32	0.574	1.52	1.05–2.19	0.027	1.07	0.81–1.41	0.629
	“Increased” vs. “unchanged”	1.04	0.63–1.71	0.882	2.20	1.40–3.47	0.001	1.53	1.07–2.19	0.019
Nuts	“Decreased” vs. “unchanged”	0.64	0.40–1.02	0.063	1.11	0.73–1.69	0.629	0.89	0.66–1.21	0.471
	“Increased” vs. “unchanged”	1.21	0.85–1.73	0.298	1.65	1.16–2.35	0.006	1.57	1.21–2.05	0.001
Dairy products	“Decreased” vs. “unchanged”	1.45	0.97–2.17	0.067	1.63	1.10–2.41	0.015	1.85	1.38–2.49	<0.001
	“Increased” vs. “unchanged”	0.98	0.64–1.48	0.915	1.01	0.66–1.54	0.960	1.26	0.94–1.69	0.126
Legumes	“Decreased” vs. “unchanged”	1.07	0.74–1.54	0.712	1.37	0.96–1.95	0.081	1.39	1.07–1.80	0.012
	“Increased” vs. “unchanged”	1.28	0.78–2.10	0.322	1.54	0.95–2.51	0.079	1.57	1.09–2.26	0.016
Water	“Decreased” vs. “unchanged”	0.93	0.60–1.43	0.727	1.25	0.82–1.91	0.291	1.12	0.83–1.52	0.461
	“Increased” vs. “unchanged”	1.37	0.96–1.95	0.080	1.69	1.19–2.39	0.003	1.52	1.18–1.97	0.001
Snacks	“Decreased” vs. “unchanged”	0.71	0.43–1.16	0.171	1.06	0.66–1.70	0.814	1.15	0.84–1.58	0.385
	“Increased” vs. “unchanged”	1.53	1.10–2.12	0.011	2.22	1.60–3.09	<0.001	1.77	1.39–2.25	<0.001
Exercise	“Decreased” vs. “unchanged”	1.20	0.87–1.66	0.262	1.18	0.85–1.62	0.316	1.44	1.15–1.81	0.002
	“Increased” vs. “unchanged”	1.50	0.90–2.48	0.117	1.48	0.89–2.45	0.130	1.69	1.17–2.46	0.006
Breakfast times	“Decreased” vs. “unchanged”	1.15	0.77–1.71	0.498	1.76	1.22–2.55	0.003	1.76	1.33–2.33	<0.001
	“Increased” vs. “unchanged”	0.80	0.39–1.62	0.534	1.21	0.63–2.33	0.575	1.03	0.63–1.67	0.909
Midnight snack times	“Decreased” vs. “unchanged”	0.90	0.58–1.38	0.622	0.97	0.64–1.48	0.900	1.12	0.83–1.50	0.472
	“Increased” vs. “unchanged”	1.08	0.62–1.88	0.796	1.04	0.59–1.82	0.899	1.17	0.78–1.76	0.454
Sleep duration	“Decreased” vs. “unchanged”	0.44	0.27–0.71	0.001	0.32	0.19–0.55	<0.001	0.35	0.26–0.48	<0.001
	“Increased” vs. “unchanged”	1.81	1.31–2.52	<0.001	2.33	1.68–3.24	<0.001	2.29	1.79–2.94	<0.001
Body weight	“Decreased” vs. “unchanged”	0.72	0.36–1.42	0.337	0.60	0.29–1.24	0.167	0.64	0.40–1.01	0.053
	“Increased” vs. “unchanged”	1.31	0.92–1.87	0.132	1.34	0.94–1.92	0.107	1.13	0.87–1.47	0.361

^1^ Multinomial logistic regression of status during lockdown on changes in eating habits and lifestyles, adjusted for age, sex, body mass index (BMI), and educational levels. Status during lockdown was grouped into 4 categories (returned to work within the first week, the second week, and the third week after the lockdown was announced, and always stayed at home/worked from home), where the group that returned to work within the first week was set as the comparison group. OR: odds ratio. CI: confidence interval.

## Data Availability

Data sharing is not applicable to this study.
